# Supplementary figures and images for: Music elicits different gene expression responses in the buccal cavity of age-related cognitive disorders patients and healthy controls
Source: Front Aging Neurosci. 2025 Sep 23;17:1622816. doi: 10.3389/fnagi.2025.1622816 (PMC12501983; doi:10.3389/fnagi.2025.1622816)

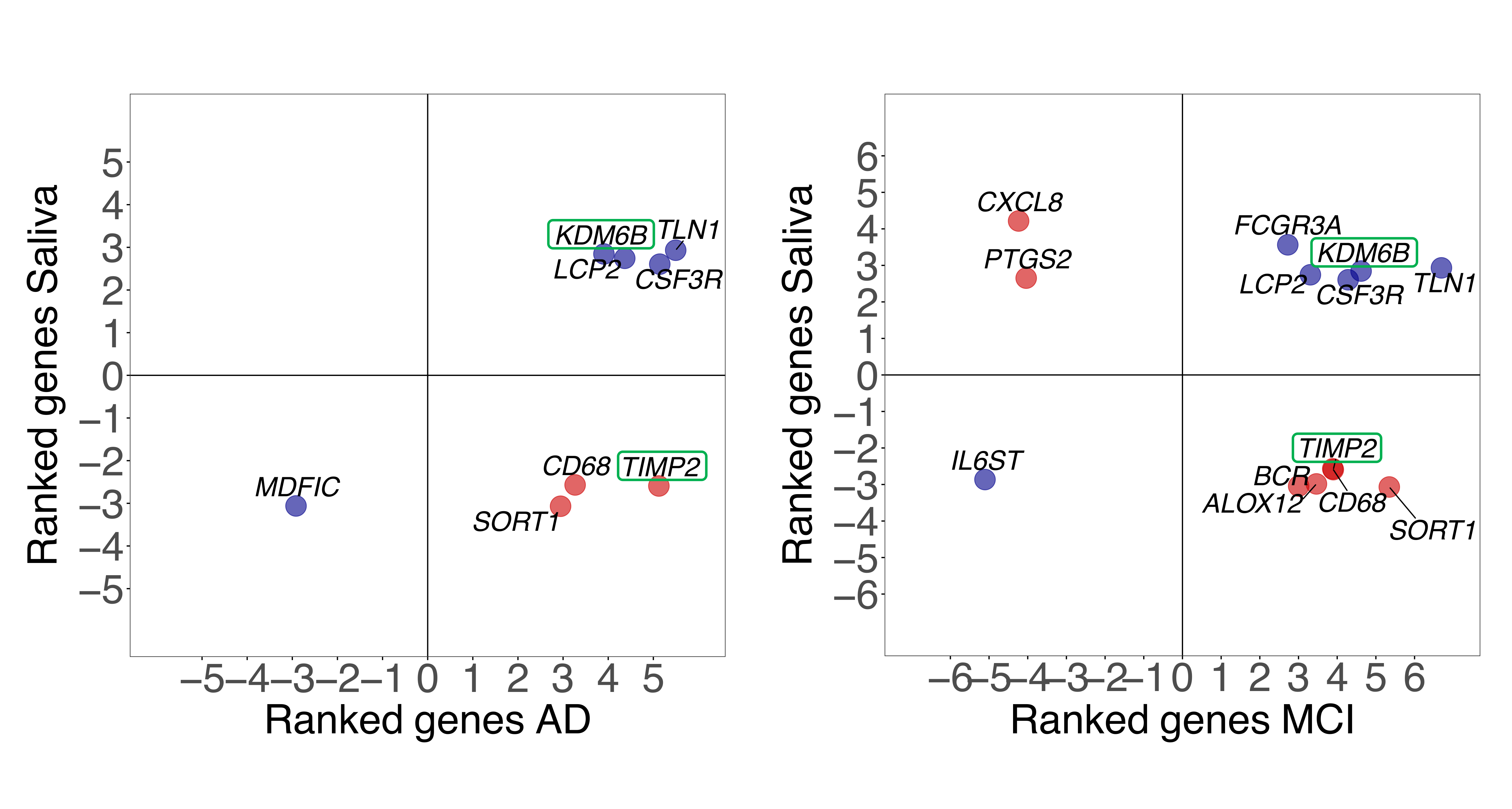

Supplement: Supplementary file 5 [file Image_1.TIF]

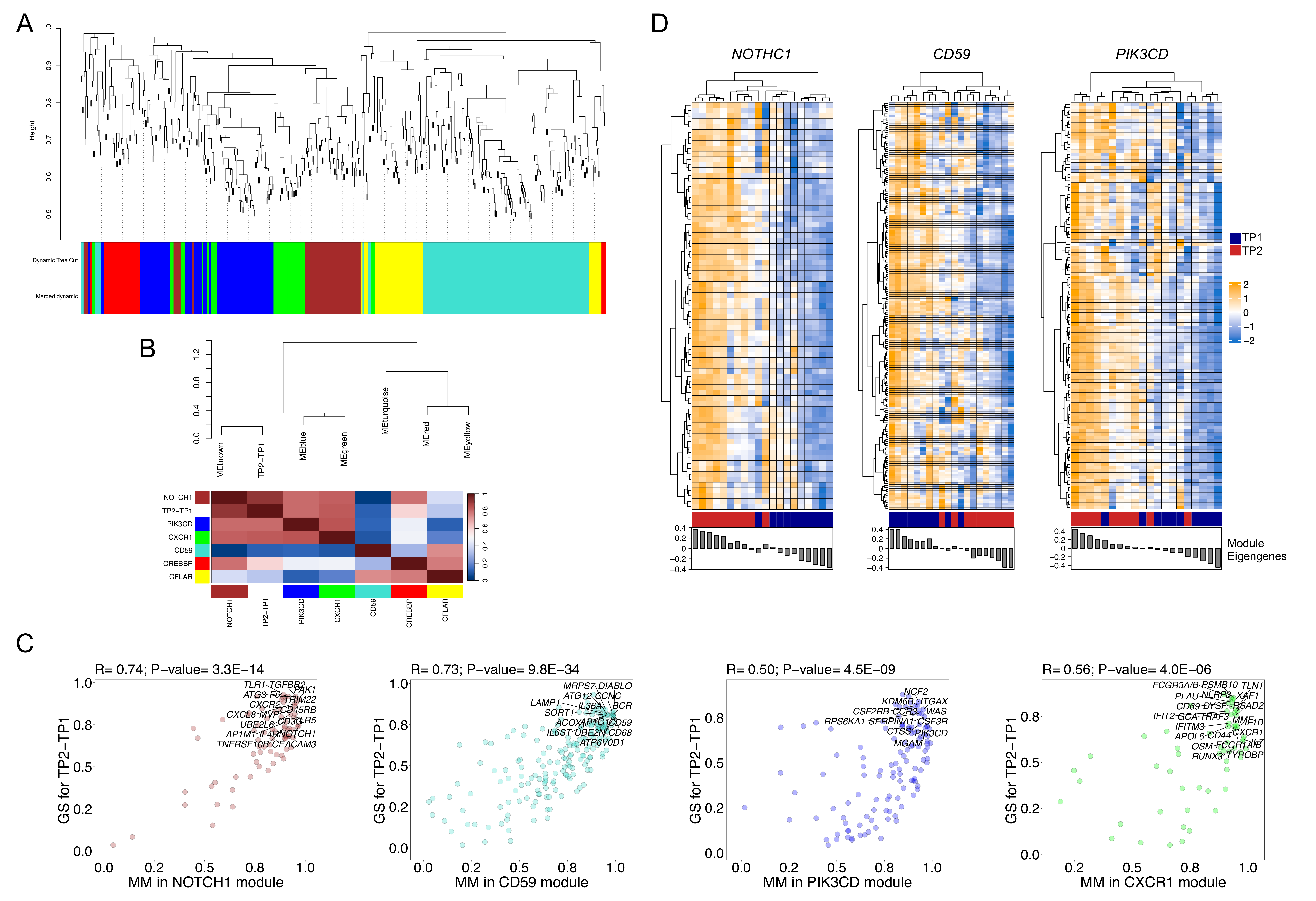

Supplement: Supplementary file 6 [file Image_2.TIF]

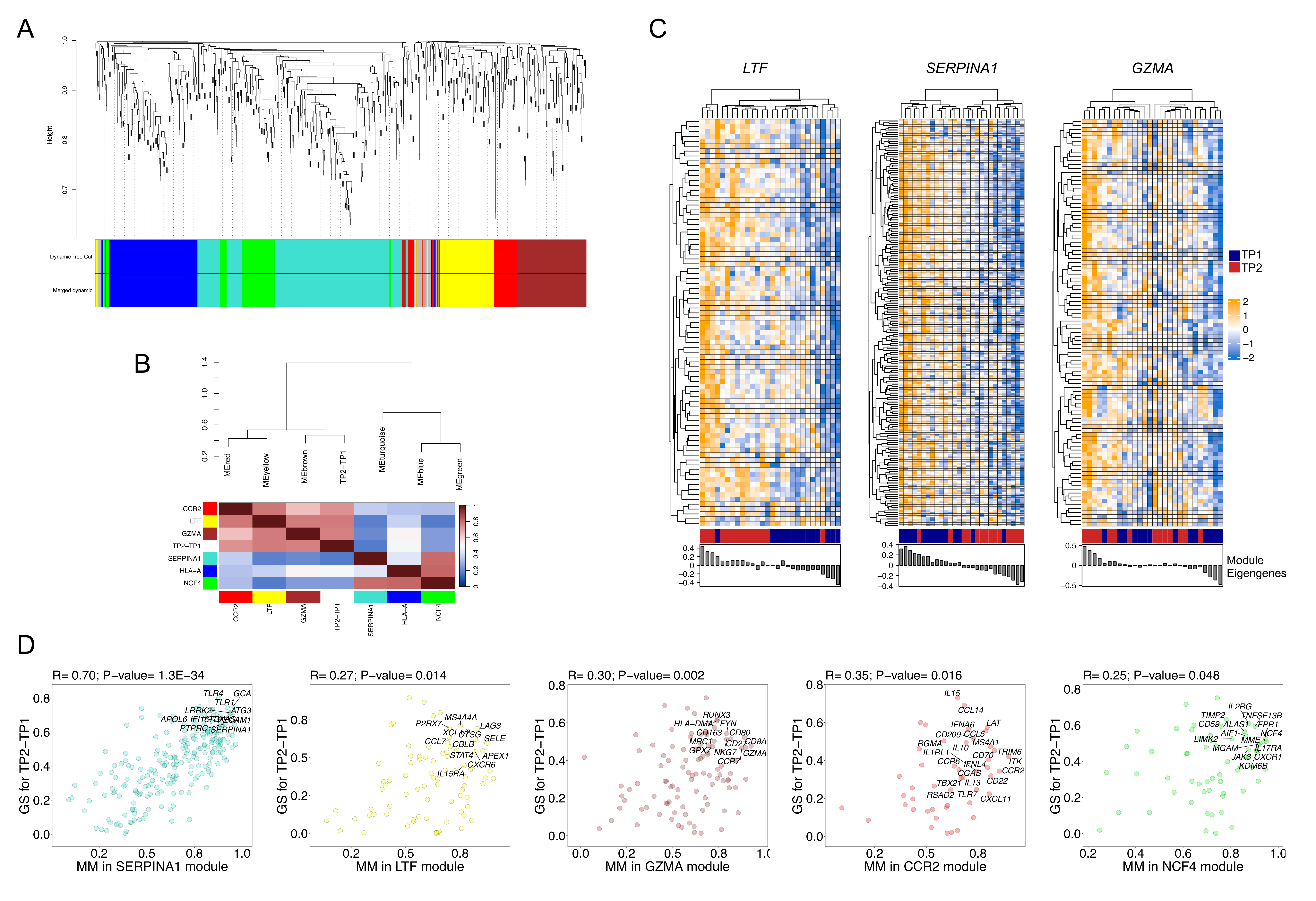

Supplement: Supplementary file 7 [file Image_3.TIF]
